# Evaluation of Mitigation Role of L-Phenylalanine-Based Low-Molecular-Weight Gelator against Oil Pollution-Induced Nile Tilapia Toxicity

**DOI:** 10.3390/gels9110848

**Published:** 2023-10-26

**Authors:** Mohamed Mehawed Abdellatif, Noha M. Sabry, Saber Ibrahim, Samah M. Bassem, Wagdy K. B. Khalil, Fagr Kh. Abdel-Gawad

**Affiliations:** 1 Chemistry of Tanning Materials and Leather Technology Department, Chemical Industries Research Institute, National Research Centre, 33 El Buhouth St. Dokki, Giza 12622, Egypt; 2 Department of Chemistry, Tokyo Metropolitan University, 1-1 Minami Osawa, Tokyo 192-0397, Japan; 3 Water Pollution Research Department, Centre of Excellence for Advanced Science (CEAS), National Research Centre (NRC), 33 El Bohouth St. Dokki, Giza 12622, Egypt; nohamsabry3@gmail.com (N.M.S.); samahbassem7@gmail.com (S.M.B.); 4 Center of Excellence for Research and Applied Studies on Climate Change and Sustainable Development, National Research Centre (NRC), 33 El Bohouth St. Dokki, Giza 12622, Egypt; wk.bassaly@nrc.sci.eg; 5 Packaging Materials Department, National Research Centre, Elbuhoth Street 33, Dokki, Cairo 12622, Egypt; sa.ibrahim@nrc.sci.eg; 6 Nanomaterials Investigation Laboratory, Central Laboratories Network, National Research Centre, Elbuhoth Street 33, Dokki, Cairo 12622, Egypt; 7 Cell Biology Department, Centre of Excellence for Advanced Science (CEAS), National Research Centre (NRC), 33 El Bohouth St. Dokki, Giza 12622, Egypt; 8 National Biotechnology Network of Expertise (NBNE), Academy of Scientific Research and Technology (ASRT), Cairo 11516, Egypt

**Keywords:** oil remediation, low-molecular-weight gelator, water quality, genotoxicity, Nile tilapia

## Abstract

A lot of oil is leaked into aquatic environments, significantly impacting fish health and, consequently, human populations. This study aimed to introduce an L-phenylalanine-based low-molecular-weight gelator (expressed as Z-Phe-C_18_) as a smart remediation tool for oil spills. Several groups of Nile tilapia were allocated in aquaria exposed to different doses of crude engine oil with/without the organogelator for 4 weeks. The results revealed a significant increase in biochemical oxygen demand, chemical oxygen demand, electrical conductivity, and total dissolved solids in water samples of fish aquaria exposed to oil pollution. The antioxidant activity levels, micronucleus formation, and expression patterns of stress-related genes were significantly higher in the livers of fish exposed to crude oil than in those of control fish. On the contrary, fish groups exposed to oil pollution and treated with the organogelator indicated that antioxidant enzymes, micronucleus incidence, and gene expression alteration of stress-related genes declined compared with those exposed to oil pollution only. The results suggest that oil pollution can induce oxidative stress via the enhancement of oxygen free radical formation. On the contrary, oil removal by the organogelator decreases oxidative stress and consequently strengthens fish immunity. So, we can conclude that organogelator treatment is promoting oxidative resistance development by increasing the activities of antioxidant enzymes, which are important in protection against oil pollution and preventing peroxidation of fish tissues. Promisingly, the organogelator could be used as a tool for the remediation of oil pollution in aquatic environments.

## 1. Introduction

The release of crude engine oil or liquid petroleum hydrocarbons into the marine environment because of increasing human activities is known as oil pollution. It poses a significant danger to marine ecology as well as coastal infrastructure. In many cases of oil pollution, waste oil, greasy ballast water, and other refined petroleum products such as gasoline and its byproducts are spilled from cargo ships. Several studies have linked the incidence of oil spills to major shipping routes, finding that spills occur often along these routes. Light volatile oil molecules quickly evaporate and oxidize, producing a highly poisonous byproduct. Heavy oils are less harmful, but they last for a long time in the environment, especially if they combine with stones and sand on beaches. Oil spills have the potential to drastically impact aquatic organisms [1]. Not only do oil spills have a great negative impact on ecosystems but so also do the emissions of lubricants via micro drops or oil mist. Bioderived lubricants from renewable sources can be a sustainable alternative to synthetic ones [2].

The behavior reactions of fish have been employed as biomarkers for contamination in the water [3]. Although adult fish are more resistant to oil pollution because of their bodies, mouths, and gill chambers, the accumulation of oil pollution is inducing fish diseases. The Nile tilapia *Oreochromis niloticus* (*O. niloticus*) is the most predominant fish species in Egypt. Industrial, agricultural, and municipal wastes are dumped into the Egyptian aquatic system, resulting in massive volumes of pollution on local fishes. Moreover, agriculture (pesticides, herbicides, and insecticides) and industrial effluents account for the vast bulk of these pollutants [4,5]. The continuation and survival of fish are threatened by the environmental pollution of water. Environmental contamination, particularly water pollution, is a severe problem that affects every country on the planet. Water pollution not only has an impact on aquatic organisms’ survival and reproduction, but it also has a negative impact on human health due to bioaccumulation [6]. Pathogenic microorganisms are increasing in water polluted with crude engine oil. The presence of harmful microorganisms in raw or insufficiently cooked seafood poses a health risk to human beings. Foodborne pathogens are normally found in aquatic habitats, as well as sewage-contaminated water that can cause health problems in fish and humans. Various bacteria, viruses, and parasites can cause seafood illnesses and intoxications. Nausea, vomiting, diarrhea, stomach pains, headache, and joint discomfort are all signs of fish food poisoning [7]. Total coliform, *E. coli*, *Salmonella*, *Pseudomonas aeruginosa* (*P. aeruginosa*), and *Staphylococcus aureus* (*S. aureus*) are pathogenic microorganisms often used to test food safety and quality. Foodborne outbreaks have been linked to the presence of these bacteria in raw fish products [8,9].

The current techniques used to treat water contaminated with oil spills include physical and chemical tools. Physical oil-spill remediation tools mainly work via the confinement of oily pollutants to prevent them from spreading or transferring to the treatment area and include foams of expanded polyurethane or polyethylene, oil skimmers, etc. [10] whereas chemical tools, such as dispersing, gelling, film-forming chemical agents, etc., depend on chemical reactions to breakdown or coat the toxic structures into inactive forms [11]. The photocatalytic approach is also an efficient technique for treating various organic dyes in aqueous solutions in an eco-friendly manner using promising photocatalysts such as AgNWs/ZnO NRs/AgNPs hierarchical nanostructures [12].

**Figure 1:**
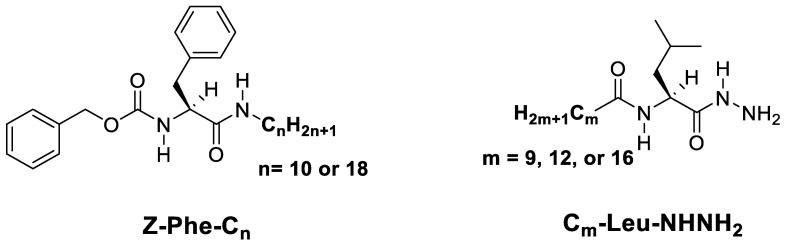
Amino acid-based organogelators. Reprinted with permission from Elsevier [20].

Low-molecular-weight gelators (LMWGs) can be considered as one of the smart chemical remediation tools. LMWGs are small molecules (less than 2000 Da) and can immobilize fluid phases (such as water, solvents, oils, liquid crystals, inorganic anions, etc.) [13,14,15,16,17,18]. This ability originates from the formation of three-dimensional fibrillar networks via supramolecular self-assembly interactions. These intermolecular non-covalent interactions of gelator molecules include, for instance, hydrogen bonding, metal coordination, van der Waals, and so on. We recently reported the synthesis of amino acid-based gelators. The designed LMWGs were prepared from L-Leucine derivatives [19] bearing different alkyl chain lengths (i.e., 9, 12, and 16) [C_m_-Leu-NHNH_2_] and L-phenylalanine derivatives with varying lengths of long alkyl chains (i.e., 10 and 18) [Z-Phe-C_n_] (Figure 1) [20]. The prepared gelators showed high gelation efficiencies and good gelation in oils, alkanes, and aromatics with low critical gel concentrations (CGC). The stacking-up (i.e., one-dimensional self-assembly process) of the organogelator molecules was investigated and referred to as head-to-tail primary stacking-up via hydrogen bonding for Z-Phe-C_n_ and C_m_-Leu-NHNH_2_ with support of π-π stacking in the case of Z-Phe-C_n_. The amino acid-based LMWGs offer many advantages such as commercial availability, eco-friendliness, biodegradability, non-toxicity, and affordable synthetic methods [21,22,23,24]. Some useful techniques can be combined to understand the gelation behavior of LMWGs such as crystallographic studies and energy frameworks analysis; applied to a series of *N*-dodecanoyl-L-amino acid derivatives, they indicate the importance of the hydrophilic parts for the stability of the gels and initiation of the gelation process, whereas the hydrophobic parts can trap the solvent molecules via tangling [25].

In this work, an L-phenylalanine-based low-molecular-weight gelator (Z-Phe-C_18_) was utilized as a smart remediation tool to remove oil spill pollution from a contaminated aquatic environment. We thus herein evaluate the remediation treatment efficiency of Z-Phe-C_18_ for polluted water in fish aquariums with different concentrations of crude engine oil. The toxicity of the crude engine oil and the potential protection effects of Z-Phe-C_18_ against oil pollution in Nile tilapia (*O. niloticus*) were assessed via chemical, microbiological, and enzyme activity analyses as well as the expression analysis of stress-related genes.

## 2. Results and Discussion

The exposure of marine organisms to environmentally toxic substances leads to genetic mutations, metabolic disorders, reduced fertility, and damage to the offspring. Thus, the use of a genotoxicity test is essential to assess the potential toxicity of marine organisms so that the risks can be controlled [26]. In the current study, physico-chemical and microbial analyses as well as several biological and genotoxicity tests including enzyme activity analysis, micronucleus test, and expression of stress-related genes were used to assess the toxic effect of crude engine oil on Nile tilapia.

### 2.1. The Synthesis of the Gelator and the Gelation Efficiency

**Figure 2:**
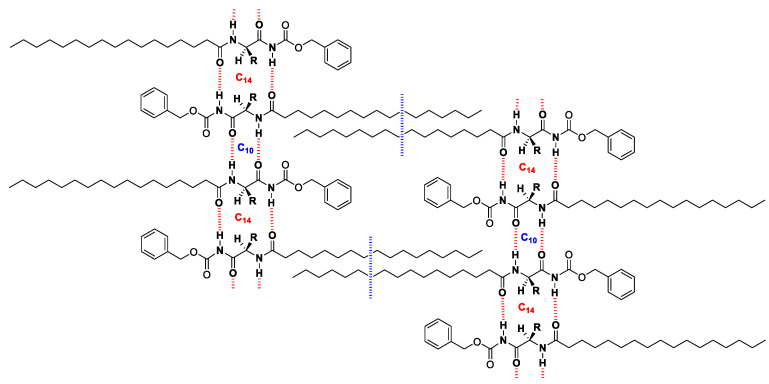
The gelation process via supramolecular interactions.

The synthesis of the L-phenylalanine-based low-molecular-weight gelator (Z-Phe-C_n_) was achieved in one synthetic step. The organogelator was prepared in high yield via the treatment of L-phenylalanine derivatives (i.e., *N*-(Carbobenzyloxy)-L-phenylalanine) with octadecyl amine in the presence of HBTU as a coupling reagent. The resultant gelator was characterized using ^1^H and ^13^C-NMR spectra (Figures S1 and S2, supplementary Material) and atmospheric pressure chemical ionization (APCI) mass spectrometry. The amphiphilic nature of the resultant organogelator acquired by the polar headgroup (i.e., amino acid) and the long hydrophobic tail (i.e., octadecyl alkyl chain) seemed to be a prerequisite for showing high gelation efficiency towards oils and alkane solvents in general. This may be driving the balance between the hydrogen bonding and the van der Waals forces (hydrophobic interactions) [22] which is necessary for the gelation process (Figure 2). The longer alkyl chain also enhances solubility, which is the primary step for gelation. The gelation efficiency was evaluated using the tube inversion testing method via wide screening using many fluids of various natures such as aromatics, cyclic alkanes, alcohols, chlorines, esters, ketones, and various oils. The critical gel concentrations (CGCs in wt%) for alkanes and oils are 0.14 (paraffin oil), 0.90 (soybean oil), 1.0 (Olive oil), 1.2 (sunflower oil), 0.11 (n-octane), and 0.52 (cyclohexane) [20]. The high gelation efficiency of the organogelator (Z-Phe-C_18_) towards oils makes it a suitable candidate as a remediation tool for oil removal.

The technique of application of the organogelator to remove spilled oil is to spray a concentrated solution of organogelator in ethanol which can convert the floating oils into a gel via phase-selective gelation [20,21,23] that can be collected easily. Three solvents can be used to prepare a solution of gelator in high concentration which are methanol, ethanol, and chloroform, but ethanol was chosen due to its nontoxicity and biodegradability, and it quickly breaks down into harmless substances if spilled [27]. The organogelator showed high phase-selective gelation of crude engine oil from its mixture with water in fish aquariums.

### 2.2. Morphological Toxicity Symptoms

**Figure 3:**
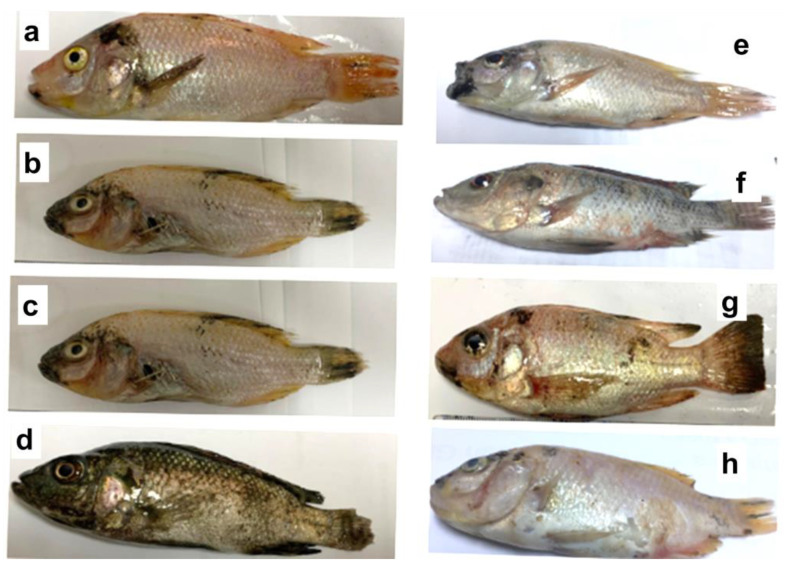
Photographs showing the effects of LMWG treatment on the color as well as growth performance of *O. niloticus* after 30 days while oil treatment shows dark skin with fin and tail rot. (**a**) Fish from the control group, (**b**) fish exposed to low dose (0.3% *v*/*v*) of oil, (**c**) fish exposed to medium dose (0.6% *v*/*v*) of oil, (**d**) fish exposed to high dose (1.2% *v*/*v*) of oil, (**e**) fish exposed to low dose of oil with gelator treatment, (**f**) fish exposed to medium dose of oil with gelator treatment, (**g**) fish exposed to high dose of oil with gelator treatment, (**h**) fish exposed to gelator only.

The results of the study indicated that the toxicity symptoms of engine oils could vary between groups according to the dose of oil. *O. niloticus* from the control and gelator groups showed normal skeletal muscles. On the other hand, fish treated with a low dose of engine oil exhibited low abnormality in the skeletal muscle, while *O. niloticus* exposed to medium and high doses of engine oil showed skin lesions that changed color or an opening in a fish*’*s skin or fins. Lesions can form on the skin*’*s surface and can penetrate deeper into a fish*’*s muscles or organs. This damaged skin might increase the penetration of toxins across the skin. However, the groups of *O. niloticus* exposed to different doses of engine oil and treated with the organogelator showed rarely noticeable damage to the skeletal muscle compared to groups exposed to engine oil only (Figure 3). The determination of the remaining oil and removal efficiencies % was difficult to obtain a precise determination of the oil contaminants in the whole fish aquarium. For that, it was more reasonable to study the consequences of crude engine oil toxicity in the exposed fish and the water quality.

### 2.3. Effect of Crude Engine Oil and Organogelator on Water Quality Parameters

The physicochemical parameters of water samples exposed to different concentrations of crude engine oil are summarized in Table 1. pH values showed a slight reduction in the pollution group when compared with the control. No definite trend was observed in the values of salinity. A significant increase in both the conductivity (EC) and total dissolved solids (TDS) of the pollution and treatment groups was observed compared with the control. Concerning the dissolved oxygen (DO), compared to the control group, there was a significant decrease (*p* < 0.05) in DO in the pollution group. However, biochemical oxygen demand (BOD), as well as chemical oxygen demand (COD), levels increased significantly (*p* < 0.05) with higher crude oil concentrations.

A significant increase in the EC and TDS of water samples of fish aquaria exposed to oil pollution was observed compared with the control. Moreover, there was a significant reduction in DO in the water samples of fish aquaria exposed to oil pollution compared with the control group. However, BOD and COD increased significantly with the increase in the concentration of crude engine oil. In the same line, Ref. [28] reported that BOD and COD values were increased due to the high hydrocarbon content of oil spills in Nile River water. This study revealed that the higher the crude oil concentration, the greater the negative effect on bacterial counts. Also, APHA [29] exhibited that total coliform and fecal coliform counts declined with increasing oil spill volume, which may be attributed to these bacteria*’*s strong sensitivity to stress conditions [29].

On the other hand, using the organogelator in the current study improved the physico-chemical parameters (EC, TDS, BOD, and COD) and microbial content when added to the oil-polluted water. In the same line, Ref. [30] indicated that low-molecular-weight organogelators have potential application for congealing oil spills to improve physico-chemical parameters and microbial content in polluted water. So, organogels could be easily used due to the following important properties: (i) easy low-cost synthesis, (ii) environmental friendliness, (iii) thermos-reversibility allowing oil recovery, and (iv) recyclability and reusability.

### 2.4. Bacterial Community Affected by Crude Oil in Nile Tilapia Fish

The assessment of microbiological parameters in the sampled fish to study the effect of three different crude engine oil concentrations is represented in Table 2. There was no statistically significant difference in bacterial contamination among the studied fish types. Total coliform and *E. coli* levels in all sampled fish of the polluted group were above the enumeration limit < 1 log_10_ [31], being the highest in high doses of crude oil (3.7 and 3.3 log_10_ CFU/g, respectively). The *S. aureus* count was below the limits listed by [32] (<2 log_10_ CFU/g) in the treatment group and, consequently, within the permissible limits for *Staphylococcus* in fish. On the other hand, *Salmonella* spp. and *P. aerginosa* were not detected in all sampled fish and, therefore, the microorganism levels were in conformity with the recommended limit of the FAO/WHO [31], except for in high doses of oil (2.6 and 2.08 log_10_ CFU/g, respectively). In general, we can note that the higher the crude oil concentration, the greater the negative effect on bacterial counts. Also, the bacterial counts reached zeros and completely disappeared in the organogelator (Z-Phe-C_18_) samples.

### 2.5. Heavy Metal Analysis of Water Samples

**Figure 4:**
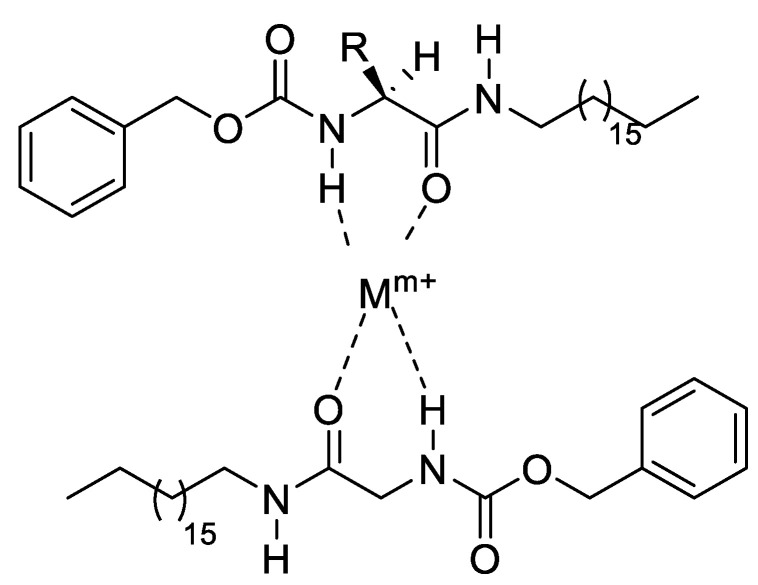
Heavy metal ion chelation with low-molecular-weight gelator (Z-Phe-C_18_).

The presence of various highly toxic heavy metals may threaten fish health in many marine environments [33,34]. The mean values and standard deviations of the distribution of heavy metals in water samples are expressed in Table 3. The levels of the heavy metal contaminant concentrations increased in water samples of high-dose oil. Also, the results showed the order of bioaccumulation of metal in water samples with high doses of crude oil to be Zn > Fe Cu > Cr > Pb> Mn and Cd > Ni > As. The low-molecular-weight gelators have a dual function as an oil remediation tool and a great ability to chelate with heavy metal ions [35,36] as shown in Figure 4. This can explain the low levels of heavy metal ions upon treatment with the organogelator Z-Phe-C_18_.

### 2.6. Biochemical Activity

**Figure 5:**
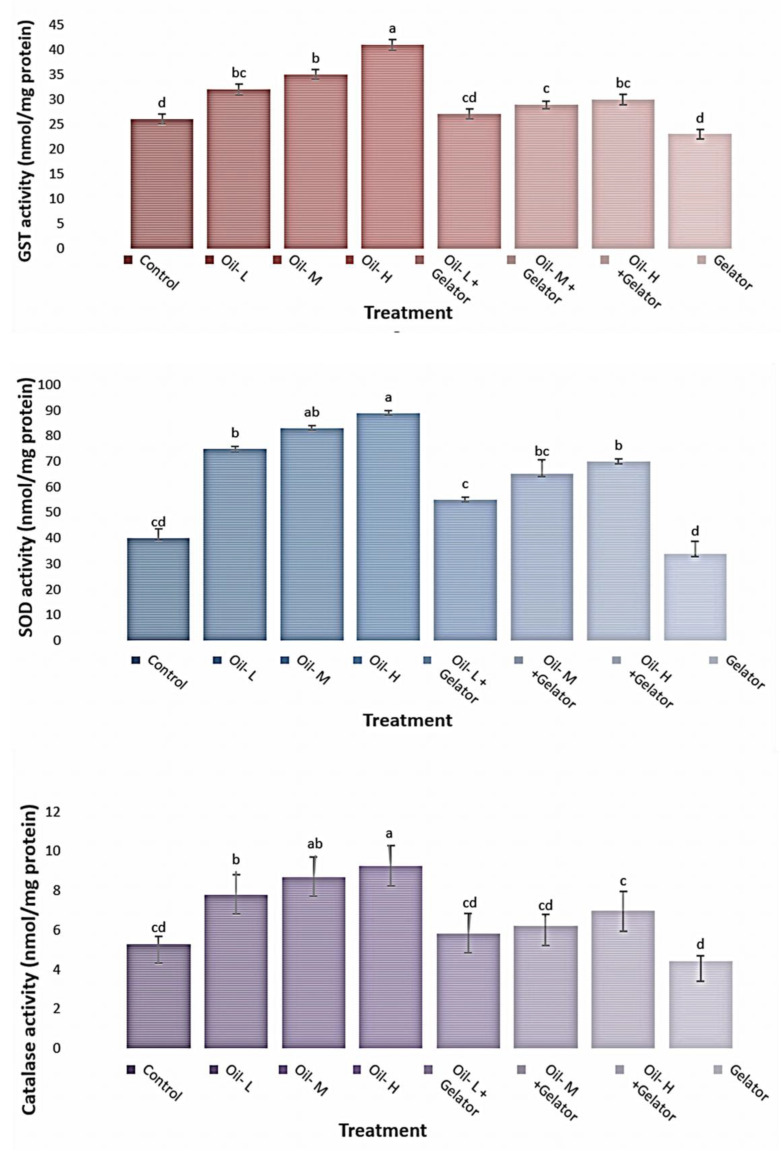
Activity levels of GST, SOD, and catalase enzymes in liver tissues of Nile tilapia exposed to crude oil in water with or without gelator. Data are presented as mean ± SEM. ^a,b,c,d^ Mean values within a row with superscript letters are significantly different (*p* < 0.05).

The results in Figure 5 show the levels of antioxidant enzymes such as GST, SOD, and catalase in the liver tissues of Nile tilapia exposed to crude oil in water with or without the organogelator (Z-Phe-C_18_). The results indicated that GST, SOD, and catalase activity levels were significantly higher in liver tissues collected from fish exposed to crude oil than in those collected from control fish. However, fish exposed to crude oil with the organogelator (Z-Phe-C_18_) exhibited significantly lower levels of GST, SOD, and catalase compared to those in fish exposed to crude oil only. Also, the treatment with organogelator (Z-Phe-C_18_) was able to elevate the levels of all three enzymes in fish exposed to oil.

In this study, the results indicated that GST, SOD, and CAT activity levels were significantly higher in liver tissues collected from fish exposed to crude oil than in those collected from control fish. In agreement with our results, [37] illustrated that prolonged exposure levels and higher crude oil concentrations enhanced the induction of SOD, CAT, and GST enzyme activities in the liver of *O. niloticus*. Furthermore, the results from numerous studies wherein goldfish were exposed to diesel oil for 40 days [38], *Prochilodus lineatus* were exposed to the same substance for 28 days [39], Atlantic cod (*Codus morhua*) were exposed to sea oil and alkyl phenol for 15 days [40], and hybrid tilapia were exposed to phenanthrene for 14 days [41] exhibited high activities of antioxidant enzymes.

Depending on the degree and period of the applied stress, and the susceptibility of the affected species, the activity of antioxidants may be increased or inhibited under chemical stress. When it comes to the metabolism of xenobiotics, the liver of fish is an organ that serves a variety of purposes [42]. For protection against ROS generated during the biotransformation of xenobiotics, hepatocytes, like other cells, rely on antioxidant enzymes [43]. This defense system*’*s enzymes include SOD and CAT, and SOD oversees the elimination of hydrogen peroxide, which is converted to oxygen and water [44]. SOD levels directly correlate with CAT activity as it is the enzyme that metabolizes superoxide radicals. So, the increase in the activity levels of antioxidant enzymes in the fish exposed to oil pollution in the current study could have resulted from high oxidative stress in the form of ROS molecules.

### 2.7. Micronucleus Test

**Figure 6:**
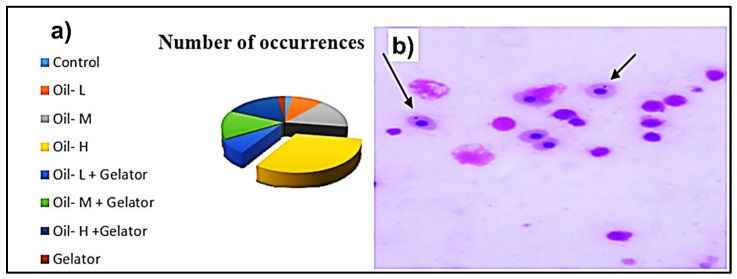
(**a**) Frequency of occurrence of micronucleated erythrocytes (MN) in gills of *O. niloticus* exposed to different concentrations of crude oil with/without organogelator treatment, (**b**) Types of micronucleated erythrocytes (MN) in gills.

The exposure of *O. niloticus* to crude oil revealed significant differences in micronuclei (MN) between exposed and non-exposed sampled fish (Figure 6). Micronuclei were low in fish exposed to crude oil with organogelator treatment. Conversely, a significant increase in the micronuclei frequency was observed in the sampled fish exposed to high doses of crude oil. However, no notable variations were detected in MN between fish that had been treated with the organogelator and the controls.

The present study noticed that exposure of Nile tilapia to crude oil revealed a significant increase in micronuclei (MN) especially with high doses of crude oil compared with control fish. In the same line, Ref. [45] observed that *Clarias gariepinus* from freshwater exposed to oil pollution exhibited a greater frequency of micronuclei chromosomal abnormalities in its genome. Additionally, Ref. [46] reported that nuclear abnormalities were identified in catfish in response to exposure to genotoxic agents. The erythrocyte count is one of the first measures to change under a stressful condition. In this investigation, it was discovered that MN rose nearly right away after the fish were moved from the control to the wasted engine oil test solutions. The majority of fish have a method for spotting polluted places, but when the entire body of water is polluted, fish must find a different route to breathe other than through their gills.

### 2.8. Expression of Stress-Related Genes

**Figure 7:**
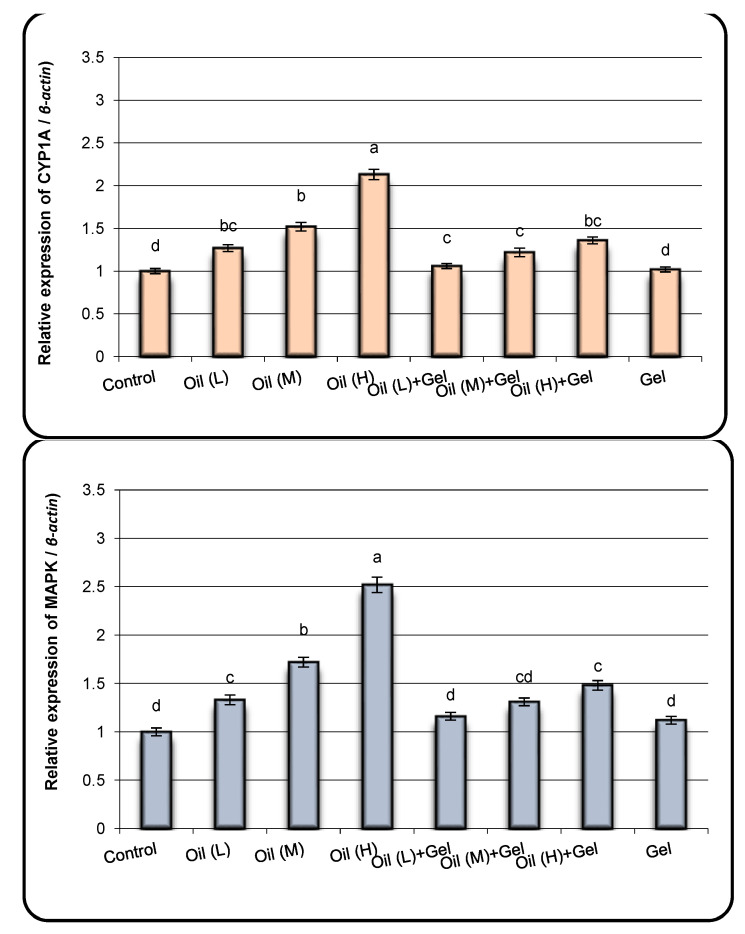

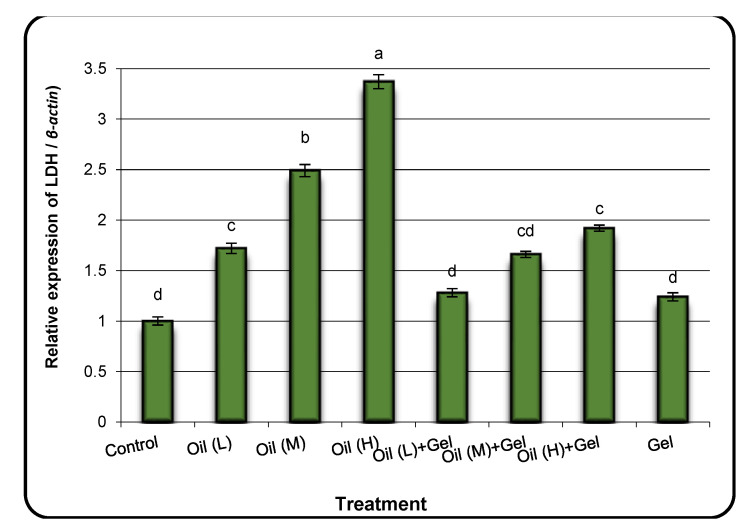
The relative expressions of Cyp1a, LDH, and MAPK genes in liver samples of fish exposed to oil and/or treated with organogelator. L: low dose, M: medium dose, H: high dose. Results are expressed as the mean ± SD. ^a,b,c,d^ Means with different letters, within tissue, differ significantly (*p* < 0.05).

The expression of stress-related genes, namely CYP1A, MAPK, and LDH genes, were analyzed in the liver tissues of fish exposed to oil and/or organogelator treatment (Figure 7). The results exhibited that fish exposed to different concentrations of oil showed over-expression of CYP1A, MAPK, and LDH genes compared with control fish. The increase in the expression levels of the previous genes was found to be highly significant at the high dose of oil. The expression levels of CYP1A, MAPK, and LDH genes were significantly decreased in groups of fish exposed to oil and treated with gel compared with those in fish exposed to oil alone especially. Moreover, the expression levels of all studied genes in fish treated with the organogelator only were relatively similar to those in control fish.

It has been observed that exposure to oil pollution causes the generation of ROS in aquatic biota. ROS have been linked to lipid peroxidation, protein breakdown, DNA damage, and changes in gene expression in fish tissues [47,48]. Changes in transcript levels are the most sensitive and early biomarkers of physiological responses to environmental stress [49,50,51]. Thus, the effect of the environment*’*s stress on aquatic organisms can be identified and quantified by employing genes whose expression levels change in response to a given environmental challenge [52,53]. The present results exhibited that fish exposed to different concentrations of oil showed over-expression of CYP1A, MAPK, and LDH genes compared with control fish. The increase in the expression levels of the previous genes was found to be highly significant at the high dose of oil. Fish groups exposed to oil pollution and treated with organogels indicated that antioxidant enzymes (GST, SOD, and CAT activity levels), micronucleus incidence, and gene expression alteration of stress-related genes declined compared with those exposed to oil pollution only. These results suggested that removing oil from the contaminated water using an organogelator decreased the oxidative stress and consequently reduced ROS formation. So, the reduction in the molecules of ROS in fish enhances the feedback mechanism of antioxidant formation and thus decreases the activity levels of SOD, CAT, and GST enzymes [54].

It is a demonstrated fact that the exposure of fish to toxins induces physiological changes in fish which include metabolic processes that are clearly linked, such as enhanced inflammatory processes, immune system modifications, and oxidative stress conditions [55,56]. So, it could probably be that oil removal using an organogelator strengthens the immune system and, therefore, decreases MN formation and reduces the expression levels of stress-related genes.

### 2.9. LMWGs as a Smart Remediation Tool for Oil Spills

LMWGs show a unique performance as smart remediation tools for oil spills. Extensive research was conducted using renewable feedstocks as building blocks such as amino acids, sugar, cholesterol, etc. These gels are able to selectively gelate various types of oil in biphasic oil–water media as shown in Table 4. In our work, we were able to introduce Z-Phe-C_18_ as an efficient smart remediation tool for the treatment of crude oil in fish aquariums and the removal of the present heavy metal ions.

## 3. Conclusions

The low-molecular-weight gelator [Z-Phe-C_18_] showed a great ability to immobilize crude oil and remove heavy metal ions in a biphasic mixture of oil–water in fish aquariums. The findings of this research associated with toxic response as well as sensitivity towards utilizing engine oils emphasize the need for greater vigilance to ensure a decrease in the risk of impairment of fish health. The toxic response of oil pollution could lead to oxidative stress by enhancing the formation of oxygen free radicals. Therefore, removing oil from contaminated water using an organogelator suppresses oxidative stress and consequently strengthens the immune system in Nile tilapia. Thus, organogelator treatment might be promoting oxidative resistance development due to increasing the activities of antioxidant enzymes and reducing the expression levels of stress-related genes, which are important markers in the protection against oil pollution preventing peroxidation of fish tissues.

The preparation of biobased LMWGs with remediation ability of oil spills and heavy metal ions is, in fact, very important in terms of improving the marine environment for a sustainable future. We strongly believe that the results could provide proof for the applicability of biobased LMWGs as a smart, nontoxic, inexpensive, and eco-friendly tool for oil-spill remediation. We hope to add more efficient examples of biobased LMWGs for various applications soon.

## 4. Materials and Methods

### 4.1. Materials

The fraction of light crude engine oil was obtained from Mobil Producing Unlimited in an airtight plastic can and transferred to the laboratory of the biotechnology and biodiversity conservation group, National Research Centre (NRC), for the current case study. *N*-(Carbobenzyloxy)-*L*-phenylalanine (Z-Phe-OH), octadecyl amine, *N*,*N*-diisopropylethylamine (DIPEA), and all other chemicals were obtained from TCI, Japan. (2-(^1^H-benzotriazol-1-yl)-1,1,3,3-tetramethyluronium hexafluorophosphate, HBTU) was purchased from Iris, Japan.

### 4.2. Analysis

The ^1^H and ^13^C NMR spectra were recorded using a Bruker AV500 spectrometer (500.13 MHz for ^1^H, 125.77 MHz for ^13^C, Bruker Japan Com., Tokyo, Japan). A Burker MicrOTOF II-SDT1 was used to perform atmospheric pressure chemical ionization (APCI, Bruker Japan Com., Tokyo, Japan) mass spectrometry.

The biotechnology and biodiversity conservation laboratory in which the biological analyses were carried out was certified with ISO 9001: 2015 for a quality management system, ISO 14001: 2018 for an environmental management system, and ISO 45001: 2018 for an occupational health and safety management system which require that the equipment and analytical methods have periodic standards to ensure the quality and accuracy of the tests and the results obtained.

### 4.3. Synthesis of Low-Molecular-Weight Gelator (Z-Phe-C_18_)

**Figure 8:**
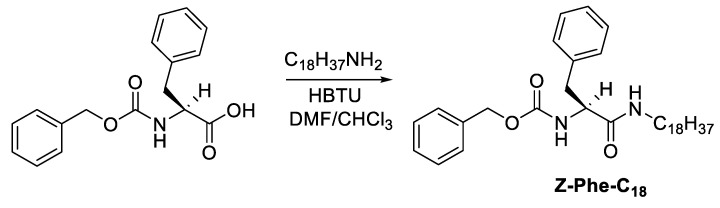
Synthesis of organogelator (Z-Phe-C_18_).

The low-molecular-weight gelator (Z-Phe-C_18_) was prepared as reported previously with a little modification [20] (Figure 8). A solution of *N*-(Carbobenzyloxy)-*L*-phenylalanine (Z-Phe-OH) (10 g, 33.40 mmol) in chloroform (100 mL), DIPEA (12.0 mL, 2.0 eq, 66.82 mmol), and octadecylamine (9.90 g, 1.1 eq, 36.74 mmol) was added to a solution containing HBTU (14.0 g, 1.1 eq, 36.74 mmol) and DIPEA (12.0 mL, 2.0 eq, 66.82 mmol) in DMF (40 mL). The mixture was then agitated at room temperature for 24 h until the reaction was finished (confirmed by TLC). Chloroform was added to the reaction mixture to dilute it (50 mL). Following that, the diluted mixture was washed twice with 5% HCl, 10% K_2_CO_3_, brine, and water. The organic phase formed was dried over anhydrous MgSO_4_. The precipitates were collected using filtration and dried in vacuo after the solids were refined by dissolving in chloroform and reprecipitating by diffusion using petroleum ether.

^1^H NMR (500.13 MHz, CDCl_3_ at 25 °C): δ (ppm) = 7.21–7.35 (br, 10H), 5.60 (s, 1H), 5.44 (s, 1H), 5.11 (s, 2H), 4.35 (s, 1H), 3.00–3.19 (m, 4H), 1.18–1.33 (br, 32H), 0.91 (t, 3H). ^13^C NMR (CDCl_3_ at 25 °C): δ (ppm) = 170.45, 155.89, 136.60, 136.16, 129.31, 128.72, 128.56, 128.23, 128.04, 127.04, 67.04, 56.53, 39.55, 38.94, 31.93, 29.71, 29.67, 29.60, 29.51, 29,37, 29.29, 29.27, 26.77, 22.69, 14.13. Yield 5.6 g (75.0%).

LRMS (APCI+) Calcd. for C_35_H_54_N_2_O_3_ [M+1]: 551.83, Found: 551.40.

### 4.4. Experimental Fish and Study Setup

Nile tilapia (*O. niloticus*) (n = 80, 20–25 g on average) were brought from the farm of the National Research Centre (Nubaria, Egypt). Nile tilapia fish were delivered in huge plastic water canisters with de-chlorinated water (24.5 °C and pH 7.2–8.2) in addition to battery aerators as an oxygen source to the laboratory of the biotechnology and biodiversity conservation group. Fish were acclimatized to laboratory conditions for one week. Following the acclimatization period, the fish were separated into eight equal-sized groups (10 fish/group) and placed into fish aquariums during the experimental period. At the end of the adaption period, the experimental fish were divided into the following groups. The first group was untreated and served as a control; fish groups from 2 to 4 contained fish exposed to different concentrations of crude engine oil as follows: 0.3% *v*/*v*, 0.6% *v/v,* and 1.2% *v/v* which served as the polluted groups [62]. The fifth to seventh groups contained fish exposed to 0.3% *v*/*v*, 0.6% *v*/*v*, and 1.2% *v/v* of crude engine oil plus treatment via spraying with a concentrated solution (1 g in 20 mL ethanol) of organogelator (Z-Phe-C_18_) in ethanol to isolate the oil via gel formation, which served as the treatment groups [63], while the eighth group contained fish treated with the organogelator (Z-Phe-C_18_) only. The experimental duration was one month, and the fish were utilized at the end of the treatment to determine the toxicity of the crude engine oil and to compare it to other groups.

### 4.5. Water Physicochemical Parameters

The pH, DO (dissolved oxygen), EC (electrical conductivity), TDS (total dissolved solids), and salinity were measured in real-time using a portable water multi-parameter detection meter (HANNA Instrument, HI98194, Hanna Instruments, Vöhringen, Germany). For 5 min, the probe was immersed in water to allow the data on the multimeter screen to stabilize. After 5 min, the findings were recorded, and the procedure was repeated for each water sample. To assess biological oxygen demand (BOD) and chemical oxygen demand (COD) characteristics, the approaches outlined in the standards for the investigation of water and wastewater [29] were used.

### 4.6. Microbiological Analysis

#### 4.6.1. Detection of Total Coliform and *E. coli*

Microbiological procedures were determined according to the ISO 16140 standard, attestation number BRD 07/7-12/04 [8]. Precisely, 10 g of fish samples were homogenized and transferred to 90 mL of buffer peptone water. To test for coliforms and *E. coli*, a sterile Petri dish was filled with rapid *E. coli* agar and one milliliter of the mixture was placed there. The plate was first incubated for 24 h at 37 °C for total coliform enumeration, then at 44 °C for 24 h for *E. coli* enumeration. Coliforms developed from blue to green colonies, whereas *E. coli* generated pink colonies as a result of this.

#### 4.6.2. Detection of *Staphylococcus aureus*

*S. aureus* was determined according to ISO 16140 and was used with the attestation number BRD 07/09-02/05 [8]. A RAPID′ Staph/Agar plate was inoculated with 0.1 mL of fish homogenate and incubated for 24 h at 37 °C. Coagulase-positive staphylococci produce black color colonies with a visible halo on the opaque medium. RAPID′ Staph/Agar, which was founded on a Baird Parker formulation optimized for S. aureus detection, enabled the counting of these species in less than 24 h.

#### 4.6.3. Detection of *Salmonella* spp.

The VIDAS^®^UP Salmonella (SPT) approved by EN ISO 16140 NF validation was used to identify *Salmonella* in fish [8]. A total of 25 g of fish was homogenized and added to 225 mL of buffer peptone water, along with 1 mL of *Salmonella* supplement. The material was then incubated at 41.5 °C for 24 h. After incubation, the sample well on the strip received 0.5 mL of enrichment broth. The strip was removed after 5 min of heating and cooled for at least 10 min. After that, the VIDAS^®^ test was carried out by immersing for 48 min the strip in the VIDAS^®^.

#### 4.6.4. Detection of *Pseudomonas aeruginosa*

*P. aeruginosa* was detected and enumerated in this study from fish according to [64,65]. After homogenizing 25 g of fish in 225 mL peptone water, samples were grown on *Pseudomonas* Cetrimide Agar (OxoidTM) and incubated at 35 °C for 48 h for observation of the distinct pigmentation.

### 4.7. Total Heavy Metals Determination

Before metal determination, all samples were digested according to standard techniques for water and wastewater analysis, where the matrix was suitable for the reliable recovery of metals that were compliant with the analytical method [66]. All heavy metal analyses (SVDV) were performed using an Agilent Inductively Coupled Plasma Spectrometer. An intensity calibration curve was created for each set of observations using a blank and three or more Merck standards (Merck KgaA, Darmstadt, Germany). To ensure the accuracy and precision of the instrument reading, Merck external reference standards, trace element standard reference material, and the National Institute of Standards and Technology (NIST) were used as quality control samples.

### 4.8. Enzyme Activity Analysis

#### 4.8.1. Glutathione S Transferase (GST) and Superoxide Dismutase (SOD) Activities

Spectrophotometric analysis was used to determine the activity of GST at 340 nm using the substrate 1-chloro-2-4 dinitrobenzene (CDNB). The expression was measured in mol/min/mg protein wet weight. The amount of SOD in the liver tissues was quantified using spectrophotometry at 480 nm using the epinephrine approach and represented in units of enzyme activities per gram of tissues wet weight [34].

#### 4.8.2. Catalase Activity

The activity of catalase (CAT) was determined using the method described by [67,68]. In a total volume of 3 mL, the reaction mixture contained 0.09 M H_2_O_2_, 0.1 M phosphate buffer, and 10% PMS. Using a double-beam spectrophotometer, changes in absorbance were measured every 30 s at 240 nm (Hitachi U-2000, Hitachi High-Technologies Corp., Tokyo, Japan). Catalase activity was measured in nmol H_2_O_2_ used per minute per g of protein.

### 4.9. Micronucleus Assay

After extraction, fish blood samples were spread on a clean surface. These slides were fixed with methanol after being allowed to dry at ambient temperature for the entire night. Using an Olympus epifluorescent microscope, smears were then stained with 0.01 percent Giemsa dye, and 2000 cells/fish were counted. For every 1000 cells, the number of micronuclei (MN) was counted [69].

### 4.10. Expression of Stress-Related Genes

#### 4.10.1. Isolation of Total RNA and Reverse Transcription (RT) Reaction

Total RNA was extracted from treated fish liver tissues using the usual TRIzol^®^ Reagent extraction procedure (Invitrogen GmbH, Darmstadt, Germany). Before usage, RNA was hydrolyzed in diethylpyrocarbonate (DEPC) water [48]. Following that, using the RevertAidTM First Strand cDNA Synthesis Kit, the entire Poly (A)+ RNA extracted from liver tissue was reverse transcribed to cDNA in a total amount of 20 µL (MBI Fermentas, St. Leon-Rot, Germany).

#### 4.10.2. Real-Time Polymerase Chain Reaction (RT-PCR)

An Applied Biosystems StepOneTM Real-Time PCR System from Thermo Fisher Scientific, Waltham, MA, USA, was used to count the copies of fish cDNA in the tissue samples. Table 5 contains a list of the particular primer sequences for the genes that were employed. To evaluate the quality of the employed primers, a melting curve analysis was carried out at 95.0 °C at the conclusion of each qPCR [47,51]. Utilizing the 2^−ΔΔCT^ method, the relative quantification of the target to the reference was established.

### 4.11. Statistical Analysis

The General Linear Models (GLM) procedure of the Statistical Analysis System [70] was used to examine the data obtained from enzyme activity analysis, micronucleus analysis, and gene expression of stress-related genes. The Scheffé test was then used to identify any significant differences between fish groups. The values were presented using mean and SEM. The probability of *p* < 0.05 was used to determine the relevance of the assertions.

## Figures and Tables

**Table 1:** Effect of crude engine oil exposure and organogelator on water quality parameters using different concentrations of crude oil expressed as mean and standard deviation.

	Control	Pollution Group			Treatment Group (with Gelator)			Gelator	Guideline CCME, 2007
		Oil (L)	Oil (M)	Oil (H)	Oil (L)	Oil (M)	Oil (H)		
pH	7.5 ± 0.2 ^ab^	7.2 ± 0.2 ^ab^	7.0 ± 0.2 ^ab^	6.0 ± 0.2 ^b^	7.9 ± 0.2 ^a^	7.6 ± 0.2 ^ab^	6.9 ± 0.2 ^ab^	8.0 ± 0.2 ^a^	6.5–9
EC (µs/cm)	236 ± 4.4 ^c^	272 ± 5.3 ^ab^	286 ± 6.7 ^ab^	292 ± 3.6 ^a^	248 ± 1.22 ^b^	260 ± 8.9 ^b^	272 ± 4.5 ^ab^	243 ± 7.8 ^c^	0.0
TDS (mg/L)	141 ± 5.3 ^d^	177 ± 6.1 ^c^	219 ± 7.3 ^b^	289 ± 1.3 ^a^	152 ± 6.2 ^cd^	163 ± 1.9 ^c^	172 ± 4.8 ^c^	146 ± 3.9 ^cd^	500
DO (mgO_2_/L)	8.0 ± 1.29 ^a^	4.8 ± 0.6 ^b^	3.8 ± 7.49 ^c^	2.9 ± 5.29 ^c^	5.9 ± 2.29 ^ab^	5.3 ± 3.8 ^b^	4.5 ± 2.6 ^b^	7.5 ± 1.9 ^a^	5.5
BOD (mgO_2_/L)	3.0 ± 0.22 ^e^	44.8 ± 0.9 ^b^	67.2 ± 0.4 ^a^	80.6 ± 0.7 ^a^	9.31 ± 1.1 ^d^	14.8 ± 1.4 ^c^	17.2 ± 0.4 ^c^	4.9 ± 0.2 ^e^	0.0
COD (mgO_2_/L)	7.0 ± 0.3 ^e^	108 ± 4.3 ^b^	117 ± 0.3 ^b^	152 ± 0.3 ^a^	31 ± 0.3 ^d^	41 ± 0.3 ^d^	83 ± 0.3 ^c^	8 ± 0.3 ^e^	7

Data are presented as mean ± SEM. ^a,b,c,d,e^ Mean values within a row with superscript letters are significantly different (*p* < 0.05).

**Table 2:** Total coliform, *E. coli*, *S. aureus*, *Salmonella* spp., and *P. aeruginosa* in sampled fish expressed as mean (log_10_ CFU/G) ± standard deviation compared to the permissible limit according to international regulations.

	Control	Pollution Group			Treatment Group (with Gelator)			Gelator	Guideline FAO/WHO 2002
		Oil (L)	Oil (M)	Oil (H)	Oil (L)	Oil (M)	Oil (H)		
Total Coliform	ND	2.5 ± 1.06 ^a^	2.9 ± 1.3 ^a^	3.7 ± 0.98 ^a^	0.52 ± 1.06 ^b^	0.7 ± 1.06 ^b^	0.9 ± 1.06 ^b^	ND	<1
*E. coli*	ND	2.0 ± 0.9	2.3 ± 0.2	3.3 ± 0.11	ND	ND	ND	ND	<1
*S. aureus*	ND	ND	2.4 ± 0.6 ^a,b^	3.5 ± 1.06 ^a^	ND	1.7 ± 1.06 ^b^	1.2 ± 1.06 ^b^	ND	<2
*Salmonella* spp.	ND	ND	ND	2.6 ± 0.3	ND	ND	ND	ND	0.0
*P. aeruginosa*	ND	ND	ND	2.08 ± 0.4	ND	ND	ND	ND	0.0

Data are presented as mean ± SEM. ^a,b^ Mean values within a row with superscript letters are significantly different (*p* < 0.05).

**Table 3:** Mean concentrations of heavy metals in water samples with or without organogelator treatment during the experimental period.

Metal Ions	Pollution Group			Treatment Group (with Gelator)			Gelator	Reference Oil Only
	Oil (L)	Oil (M)	Oil (H)	Oil (L)	Oil (M)	Oil (H)		
As	<0.001	<0.001	<0.001	<0.001	<0.001	<0.001	<0.001	0.1
Cd	0.02	0.02	0.03	<0.001	<0.001	<0.001	<0.001	0.07
Cr	0.02	0.02	0.04	<0.01	<0.01	<0.01	<0.001	0.25
Cu	0.02	0.06	0.1	0.01	0.02	0.03	<0.001	1.4
Fe	0.12	0.37	0.6	<0.01	<0.01	0.02	<0.001	28
Mn	0.01	0.01	0.03	<0.01	<0.01	<0.01	<0.001	0.35
Ni	0.01	0.01	0.02	<0.01	<0.01	<0.01	<0.001	0.2
Pb	0.02	0.03	0.05	0.01	0.01	0.02	<0.001	0.35
Zn	1.06	2.24	3.5	0.02	0.03	0.06	<0.001	266

Control is <0.001, all concentrations in mg/L.

**Table 4:** Biobased low-molecular-weight gelators are used in biphasic oil–water mixtures.

Starting Material	Pollutant	Reference
*N*-Lauroyl-L-alanine ^a^	kerosene, petrol, and paraffin	[35]
Sugar [D-(+)-Mannitol and D-(+)-Sorbitol] ^b^	diesel, mineral oil, silicone oil, and crude oil fractions	[57]
Cholesterol ^c^	xylene and/or kerosene	[58]
*N*-Acetylglucosamines ^d^	petrol, kerosene, diesel, silicone oil, and pump oil	[59]
Monoglyceride ^b^	diesel and kerosene	[60]
L-Valine and L-Isoleucine ^a^	salad oil	[61]
L-Phenyl alanine ^b^	crude oil and heavy metal ions in fish aquariums	This work

Method of application: ^a^ Heating, or in ethanol. ^b^ In ethanol. ^c^ By mixing. ^d^ In THF.

**Table 5:** Primer sequences used for qRT-PCR.

Gene	Primer Sequence (5′–3′) *^a^*	NCBI Reference
MAPK *^b^*	F: ATTCAGAGGGTGGGAAGTGG R: GTGCTTGCATTCCTTCACCA	XM_003449968.5
LDH *^c^*	F: CGAAAGCCGTCTCAATCTGG R: CAGAGTCGAGGTTAGTGCCA	EU313200.1
CYP1A1 *^d^*	F: TAAACTGCAGAGCGAGAGCA R: CTTTCGACCCCAGATAACCA	XM_019365994.2
*β*-Actin *^e^*	F: CCAGCCTTCCTTCCTTGGTA R: AGGTGGGGCAATGATCTTGA	KJ126772.1

*^a^* F: forward primer; R: reverse primer. *^b^* MAPK: mitogen-activated protein kinase. *^c^* LDH: lactate dehydrogenase. *^d^* CYP1A1: Cytochrome P450 Family 1 Subfamily A Member 1. *^e^ β*-Actin: beta-actin.

## Data Availability

The data presented in this study are openly available in article.

## Supplementary Materials

The following supporting information can be downloaded at: https://www.mdpi.com/article/10.3390/gels9110848/s1, Figure S1: ^1^H NMR chart of Z-Phe-C_18_ (in CDCl_3_ at 25 °C); Figure S2: ^13^C NMR chart of Z-Phe-C_18_ (in CDCl_3_ at 25 °C).
