# Supplementary material for: Evaluation of Mitigation Role of L-Phenylalanine-Based Low-Molecular-Weight Gelator against Oil Pollution-Induced Nile Tilapia Toxicity
Source: Gels. 2023 Oct 26;9(11):848. doi: 10.3390/gels9110848 (PMC10670902; doi:10.3390/gels9110848)
Supplement: Supplementary file 1 [file gels-09-00848-s001.zip › gels-2623817-supplementary.pdf]

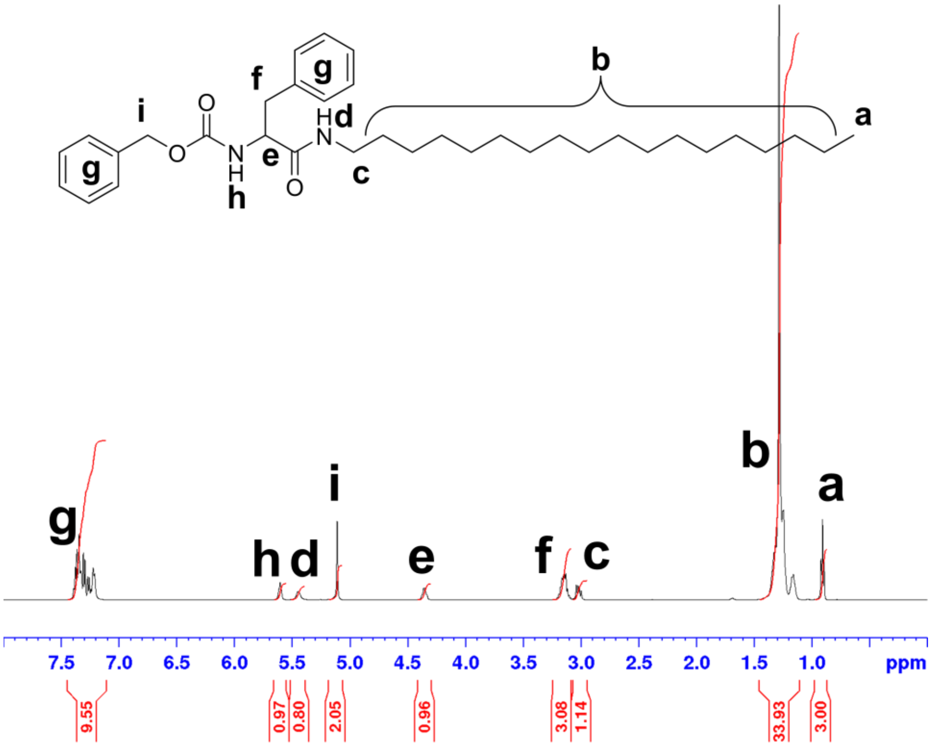

Fig. S1. ^{1}H NMR chart of Z-Phe-C_{18} (in $CDCl_3$ at 25°C).

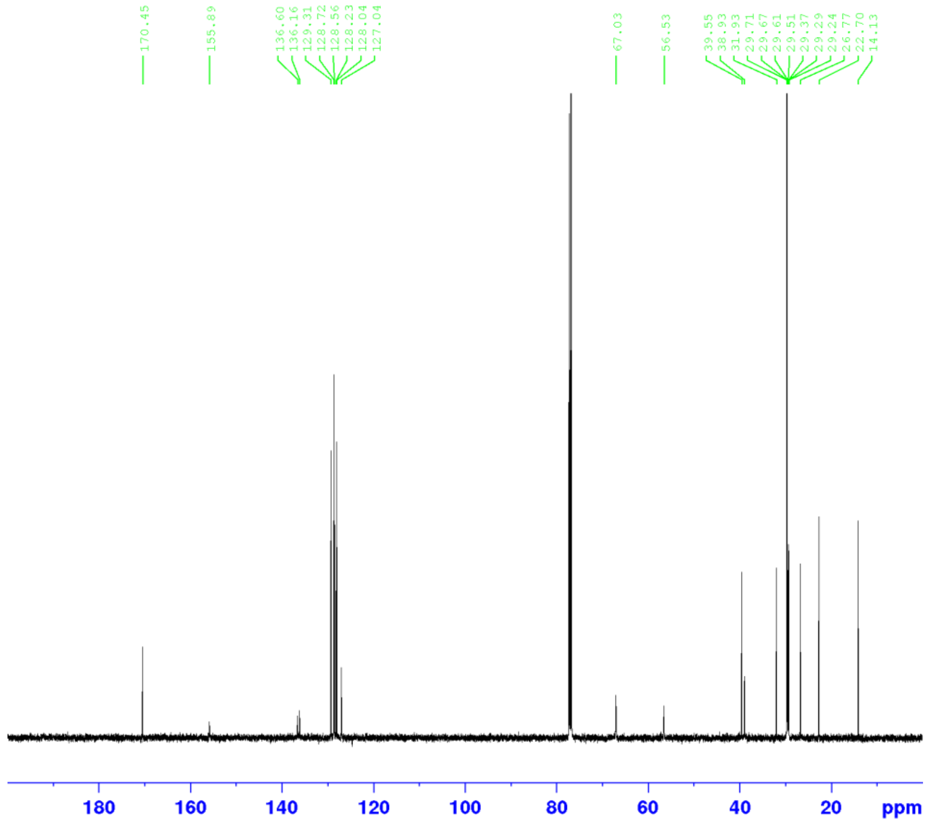

Fig. S2. ^{13}C NMR chart of Z-Phe-C_{18} (in $CDCl_3$ at 25°C).
